# Morphogengineering roots: comparing mechanisms of morphogen gradient formation

**DOI:** 10.1186/1752-0509-6-37

**Published:** 2012-05-14

**Authors:** Verônica A Grieneisen, Ben Scheres, Paulien Hogeweg, Athanasius F M Marée

**Affiliations:** 1 Computational & Systems Biology, John Innes Centre, Norwich Research Park, Norwich, NR4 7UH, UK; 2 Molecular Genetics Group, Dept. of Biology, Utrecht University, Padualaan 8, Utrecht 3584 CH, The Netherlands; 3 Theoretical Biology Group, Dept. of Biology, Utrecht University, Padualaan 8, Utrecht 3584 CH, The Netherlands

## Abstract

**Background:**

In developmental biology, there has been a recent focus on the robustness of morphogen gradients as possible providers of positional information. It was shown that functional morphogen gradients present strong biophysical constraints and lack of robustness to noise. Here we explore how the details of the mechanism which underlies the generation of a morphogen gradient can influence those properties.

**Results:**

We contrast three gradient-generating mechanisms, (i) a source-decay mechanism; and (ii) a unidirectional transport mechanism; and (iii) a so-called reflux-loop mechanism. Focusing on the dynamics of the phytohormone auxin in the root, we show that only the reflux-loop mechanism can generate a gradient that would be adequate to supply functional positional information for the *Arabidopsis* root, for biophysically reasonable kinetic parameters.

**Conclusions:**

We argue that traits that differ in spatial and temporal time-scales can impose complex selective pressures on the mechanism of morphogen gradient formation used for the development of the particular organism.

## Background

Biological development is characterised by growth and differentiation of cells, which through cell divisions, cell shape changes and cell displacements ultimately shape and form an organism. To establish different tissues, both cell fate (the commitment to differential developmental programs) and cell differentiation (the actual modifications of the cell’s biochemical and biophysical properties) are essential. “Positional information” provides cells with directions for fate changes or cell differentiation due to the position of the cell within the embryo or tissue^a^. Within this context, Turing [1] coined the term “morphogen” to describe molecules whose spatial distribution within the organism determines patterns of gene expression as cells respond to differences in the concentration. He demonstrated the feasibility of *de novo* establishment of non-homogeneous morphogen patterns, arising without the need of pre-patterns such as localised sources or sinks. In contrast, Wolpert [2] argued that it is reasonable to assume pre-patterns, for example due to maternal factors or previously established cell polarity. He showed that a combination of pre-localised sources or sinks, diffusion of the morphogen and overall decay can result in a graded morphogen distribution that could supply positional information, using threshold concentrations, that is more instructive than the original pre-pattern: the French flag model. Since then the concept of morphogens has formed a common framework to test and understand aspects of animal and, more recently, plant development [3-7].

Alternative mechanisms for cell fate changes and cell differentiation depend on cell history [8,9], local interactions [10-14] and mechanical effects [15-18]. While alternative mechanisms were proposed, the relative importance of morphogen gradients and positional information was questioned [19,20]. The core of the criticism is that it is not trivial to establish a stable, noise-resistant and accurate gradient spanning sufficiently relevant distances over the embryo, solely through production, degradation and diffusion of a morphogen alone [19,21-28].

For a number of biological systems it has been convincingly shown that graded concentrations of proteins bring forth multiple developmental outcomes. For example, in the *Drosophila* embryo it has been directly measured that Bicoid (Bcd), Hunchback (Hb), Hedgehog (Hh), Decapentaplegic (Dpp) and Wingless (Wg) form morphogen gradients and determine precise positional information [29-34]; in *Xenopus*, activin forms a gradient and acts in a dose-dependent manner [4,35]; and in the chick, a morphogen gradient of Sonic hedgehog is involved in neural tube and limb bud development [36], while a gradient of FGF8 plays an important role in somitogenesis [37].

Classical and modern studies on plant development (e.g. [38-40]) indicate that the phytohormone auxin is directly or indirectly involved in regulating virtually every spatially organised aspect of plant development. Moreover, different dosages of auxin have been shown to generate variable developmental outputs, although cellular context is a determinant as well [41-46]. Localised accumulation of auxin activates both meristematic activity and the formation of new apical primordia [47-49]. The establishment and maintenance of the longitudinal pattern of the root meristem has been inferred to be controlled by a graded auxin distribution, while interferences with this gradient lead to dramatic patterning defects in the root [50-53]. Moreover, measurements on IAA accumulation in specific cell types in the root meristem have provided a more direct support for the existence of an auxin gradient with a distinct maximum in the organising centre of the root tip [54]. Thus, at least in the context of the root meristem a graded auxin distribution is essential for cell specification.

**Figure 1:**
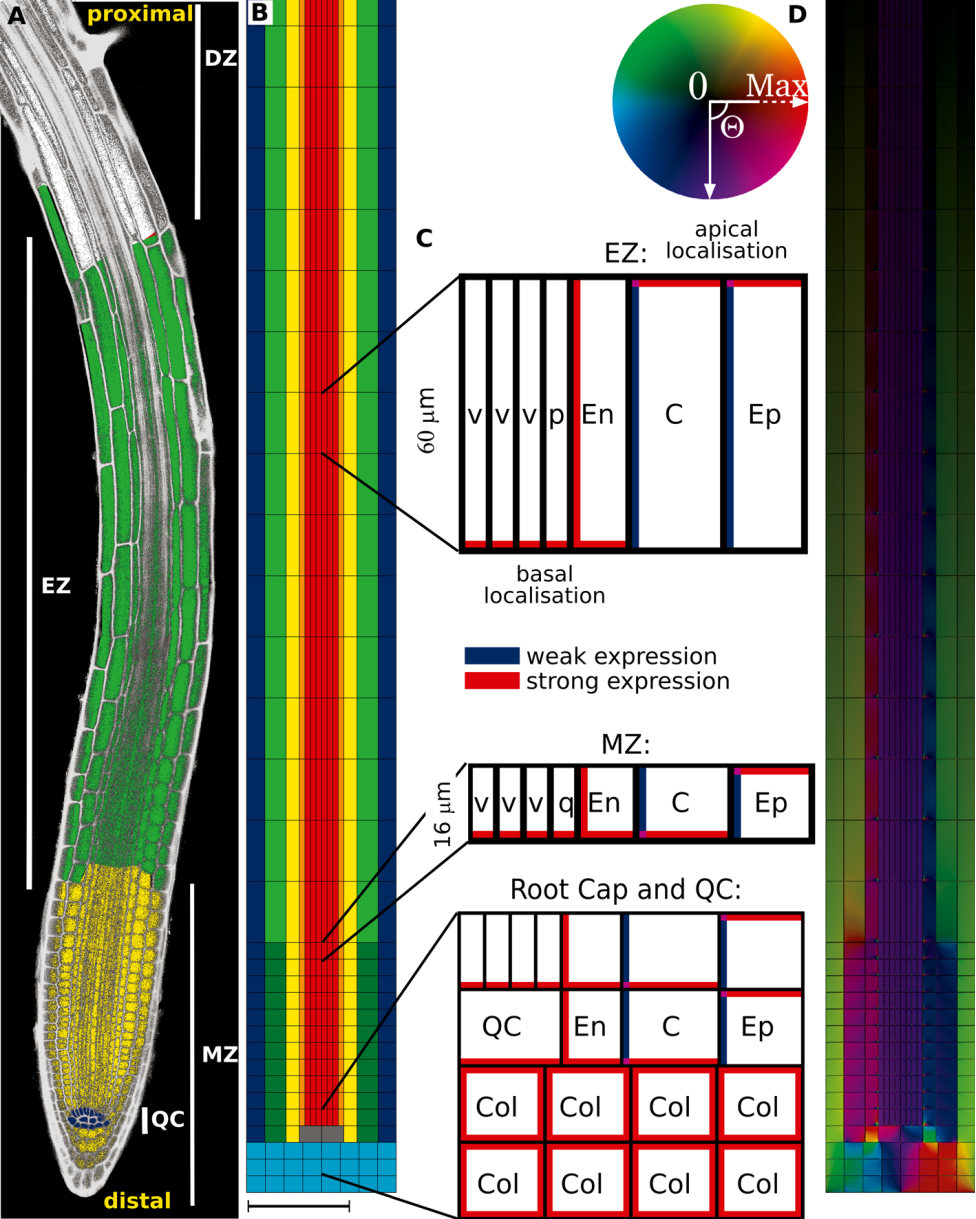
**The root and the PIN-mediated reflux-loop.** (**A**): Cross-section of a root containing the stem cell niche and quiescent centre (QC) (blue), and the meristem (MZ, yellow), elongation (EZ, green) and differentiation (DZ, white) zone. (**B**): *In silico* root segment used for simulations (here the most distal 1200 *μ*m is shown of the simulated 3 mm long segment). The distinct cell types treated in the simulations are: vascular, red; pericycle, orange; endodermis, yellow; cortex, green (dark in MZ, light in EZ); epidermis, blue; quiescent cells, grey; and columella tiers, cyan. A cell wall/apoplast (black) of 0.5 *μ*m surrounds all cells. Cell lengths change for all cell types from 16 *μ*m in the MZ, to 60 *μ*m in the EZ. Cell widths differ slightly, according to experimental images. Note that for the unidirectional transport mechanism, only the vascular and pericycle tissue are considered, together with the QC. (**C**): PIN localisation is specified in a tissue-dependent manner, based on experimentally observed distributions [see [58]]. PIN-mediated permeability follows the observed PIN expression levels, and was set to either _*Ppin,w*_=5*μ*m/s (corresponding to ‘weak’ expression levels, indicated in blue), or _*Ppin,s*_=20*μ*m/s (‘strong’ expression, indicated in red). Where PINs are not observed experimentally, a background permeability of _*Pbg*_=1*μ*m/s is assumed. Diffusion occurs within cells with a default value of 600 *μ*m^2^/s, and in the cell wall with a 15-times reduced coefficient of 40 *μ*m^2^/s. Influx is considered to be apolar in all cells, with _*Paux*_=20 *μ*m/s. (**D**): Resulting auxin reflux-loop through the root tissue. Colours show direction and magnitude of fluxes, as indicated by the colour circle to the left. For more details regarding choice of root layout, expression levels and parameters, see [53] and [58]. Scale bar 100 *μ*m (**A,B,D**).

PLETHORA (PLT) genes encode auxin-inducible transcription factors expressed in roots, which have been shown to be essential for determining differentiation in a graded manner [55,56]. PLT protein levels correlate with the auxin response gradient at the root. High levels of PLT activity are required for stem cell niche identity and maintenance, intermediate levels are essential for cell growth and proliferation in the meristem zone (MZ), and low levels are needed for cell expansion in the elongation zone (EZ) and allow further cell differentiation in the differentiation zone (DZ) [56]. Although PLT gene expression is auxin dependent, other factors contribute to the shape of the PLT gradient [57]. The three major transitions, between Stem cell niche/MZ, MZ/EZ and possibly EZ/DZ, resemble the idealised separation of colours in a flag (Figure 1A). The root, however, rapidly grows, and unlike most animal systems, in which the gradient specifies zones of different cell fate, here most cells transiently ‘move through’ the different zones due to the root tip moving deeper into the soil. Auxin thus transfers information to move to a next phase of cell differentiation rather than a different cell fate.

Previously we have shown that the mechanism which generates the auxin gradient within the distal root of *Arabidopsis thaliana* differs from earlier studied morphogen-gradient generating mechanisms [53]. Here we ask whether it would be possible for the root to have evolved an alternative mechanism for gradient establishment. We analyse and contrast three distinct mechanisms of morphogen gradient formation: (1) source-decay mechanism: morphogen production at a localised source and overall decay, the classical mechanism as proposed by Wolpert [2]; (2) unidirectional transport mechanism: directed transport of the morphogen into the direction of a ‘dead end’, where a maximum will be formed, as proposed by Mitchison [59]; (3) reflux-loop mechanism: a combination of a downward and upward flux, linked to each other through a lateral flux, forming an ‘auxin capacitor’, as proposed in Grieneisen et al. [53]. In the spirit of Lander [20], “Sometimes, answering the most qualitative of questions – ‘Why does the organism do it that way?’ – succeeds only through the most quantitative of approaches”, we will contrast the three mechanisms quantitatively, to gain insights on diverse morphogen gradients in terms of spatial and temporal scales and the implications of their differences to development.

### General concepts of morphogen gradients

**Figure 2:**
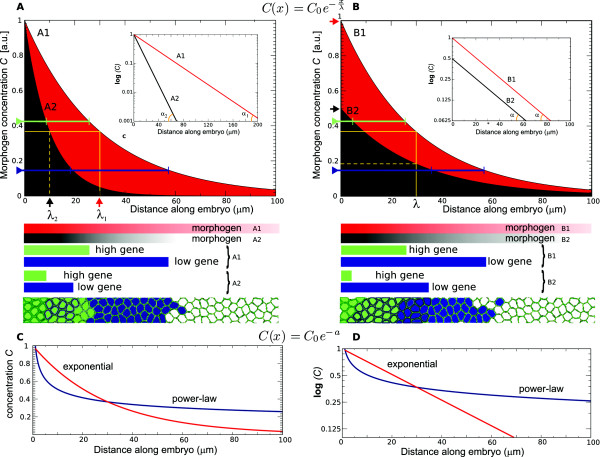
**Properties of morphogen gradients and positional information.** (**A**): Morphogen concentrations as a function of distance along a tissue, represented by exponentially decreasing profiles. Red (A1) and black (A2) profiles differ with respect to their characteristic lengths, _*λ*1_=30 *μ*m (red arrow) and _*λ*2_=10 *μ*m (black arrow), respectively, which on a log-linear plot (inset) corresponds to the inverse of the slope of the morphogen profiles ($\lambda =\frac{1}{\alpha }$; with slope _*α*1_for A1 and _*α*2_for A2). Positional information is conveyed through the graded distribution of morphogens, by means of concentration thresholds that activate different genes (‘high’ and ‘low’ gene thresholds, indicated by the green and blue lines). The spatial range of the gene expression within the tissue is affected by *λ*, as is schematically shown beneath the graph, causing differential gene activation regions. Differences in gene expression in its turn steers differentiation of a field of cells, reacting in an equivalent manner to the morphogen, into different regions, as schematically indicated in the lowermost panel. (**B**): Exponential gradients can also differ with respect to the maximum concentration (_*C*0_): even when the *λ*’s of two morphogen gradients (B1, B2) are equivalent (_*λ*1,2_=*λ*=30), the maximum concentrations (here, _*C*0,*B*1_=1, _*C*0,*B*2_=0.5) influence the positional information experienced by the tissue. This is due to the dependency of the gene expression on the absolute morphogen concentrations, as depicted in the schematic drawing below. (**C**): Comparison between an exponential (red) and a power-law morphogen profile (blue); both profiles have the same concentration value at the characteristic length of the exponential gradient (*λ*=30). (**D**): The exponential gradient presents a linear profile in the log-linear representation, whereas the power-law profile has relatively higher values at larger distances from the maximum (i.e. the profile has a long-tail distribution).

Morphogen gradients should be able to transfer positional information to all the cells within the relevant developing tissue. Thus, concentrations should vary sufficiently from one region within the tissue to another, such that cells are able to distinguish between different locations and unleash appropriate diverging (genetic) responses (Figure 2A,B). Moreover, absolute concentration values should not become too low. For all three mechanisms that we explore and contrast here, the morphogen distribution in space takes the form of an exponential gradient (see introductions on the different mechanisms below). Consequently, the distance over which positional information can be transferred in a meaningful way can be partly estimated using the slope of the gradient on a logarithmic scale (see Figure 2C,D). Likewise, the characteristic length of a morphogen gradient, *λ*, indicates the distance from the location of maximum concentration, _*C*0_, at which the concentration has fallen to _*C*0_/*e*(37%) of the maximum value. This can directly be related to the logarithmic slope of the gradient (see Figure 2).

The characteristic length defines an ‘information scale’ for the gradient, and should be in accordance to the scale of the tissue. Consequently, *λ*is tightly linked to the functionality of the gradient, given the size of the system. For example, in *Drosophila* development, the size of the system, *L*, is the total length of the embryo, roughly 480 *μ*m, while the characteristic length of the Bcd gradient is 120 *μ*m. Thus, both length scales are in the same ball park. This is important, because if the characteristic length were too long (*λ*≫*L*), positional information would be smudged out due to fluctuations. In contrast, if the characteristic length were too small (*λ*≪*L*), a large fraction of the tissue would experience very low morphogen concentrations, which poses a problem, because at low molecule numbers unavoidable side-effects related to intrinsic noise start to dominate, impeding positional information [60,61]. (Although it remains the case that it is not theoretically impossible to extract positional information from low concentration regions, by means of time averaging of the concentration variations [62].) The ratio of the length scale of the region of interest over the characteristic length of the morphogen gradient, *L*/*λ*, is called the Thiele modulus, a direct indicator of the functionality of the gradient [63]. A source-decay model with quadratic decay has been specifically proposed to reduce the sensitivity for intrinsic noise [64], (Figure 2C,D). In this study, however, we focus on exponential morphogen distributions, which is the typical distribution found in experiments [5,26,65].

Also the maximum concentration _*C*0_influences the positional information. This is because when the slope of the distribution is fixed, it is _*C*0_ that determines the distance along the tissue at which concentrations stay above the given threshold for a certain gene activation (Figure 2B). And given a minimum threshold value that can be read out robustly, a lower value of _*C*0_ thus implies a smaller maximum distance at which the morphogen gradient can exert an effect. At the same time, a higher value of _*C*0_implies higher maintenance costs for the gradient, so increasing functionality comes with a price.

Most models concerning morphogens are assuming that patterns are driven by steady state gradients. However, during development, morphogen establishment and downstream signalling and genetic responses do not politely wait for one another. A more likely situation is that while morphogen distributions are still varying over time, cells already respond. To evaluate the functionality of a morphogen gradient we should therefore analyse the temporal dynamics as well. Not only the time-scale of gradient establishment, but also the time-scale on which the gradient can be modified, when this is for example required due to growth of the tissue itself.

## Results and discussion

### Generating a morphogen gradient with auxin

#### Auxin kinetics

The phytohormone which forms a gradient along the root, indole-3-acetic acid (IAA) or simply auxin, has a molecular weight of only 0.175 kDa. Measurements on auxin diffusion show that the diffusion coefficient in the cytosol would be as high as 300–600 *μ*m^2^/s, while it would be around 15 times lower in the cell wall [66-69], i.e. 20–40 *μ*m^2^/s. Note that these values are orders of magnitude higher than the diffusion rates of the proteins with much higher molecular weight that have been carefully measured in *Drosophila* embryo and wing disc (Wg: 0.05 *μ*m^2^/s; Dpp: 0.1 *μ*m^2^/s [65]; Bcd: 0.3–17 *μ*m^2^/s [70,71]).

Outside the cell, auxin is in a neutral state and can easily permeate across the plasma membrane (PM) into the cell; due to the higher pH in the cytosol, however, once entered it becomes anionic, and its passage out of the cell becomes much more difficult [72]. However, auxin efflux and influx carriers, most importantly PINs and AUX1, increase the flux of auxin in both directions across the PM [39,73-75], (Figure 1B,C). Efflux carriers, when polarly localised along the PM and in the same polar fashion along a file of cells, are able to generate directed auxin fluxes through the plant tissue, which is taken into account in the unidirectional transport and reflux-loop mechanisms (Figure 1D, see below).

Although there is also production in the root, auxin biosynthesis predominantly takes place in the shoot, after which it is transported towards the root. For all three mechanisms we assume a constant auxin increase in the root (through influx or production), arbitrarily set to 1000 a.u./s [see discussion in [53]]. Root cut experiments have shown that without auxin influx a (slowly retracting) gradient can be preserved for many days up to weeks, indicating the presence of a very slow (net) auxin decay. Based on these experiments, we use an effective decay parameter value of 1^0−6^/s, corresponding to a half life of 8 days. Again, this is much slower than values found in *Drosophila* (Wg: 1.4×1^0−5^/s; Dpp: 2.5×1^0−4^/s [65]).

The length scale over which the auxin gradient is relevant for *Arabidopsis* root development, *L*, is around 500 *μ*m–1.5 mm, which is a bit larger than found for *Drosophila* (embryo: *L*≈480 *μ*m; wing disc: *L*≈100 *μ*m). The characteristic length of the auxin gradient is not well-established, but can indirectly be inferred to be around 100–200 *μ*m, by combining insights from coarse-scale auxin measurements [54], the PTL gradient [55,56], zonation [76] and modelling [53]. This is comparable to the Bcd gradient in the *Drosophila* embryo (*λ*≈100–120 *μ*m [26]), but much larger than what has been found in the wing disc (Wg: *λ*≈6 *μ*m; Dpp: *λ*≈20 *μ*m [65]).

#### Source-decay mechanism

When considering the spatial distribution of morphogens over space, the most standard mechanism is that of morphogens being produced at a localised source, diffusing and being degraded. We first ask what type of morphogen distribution is to be expected in a plant system when a source-decay mechanism would be at work. Answering this question is important to obtain a clear picture of what kind of positional information a plant can establish without using polar transport. In particular, a source-decay system which is determined by a linear decay is arguably the most simple gradient-generating mechanism that is used in biological development. In such a system, the dynamic changes in the morphogen concentration can be described mathematically as

(1a) $$\frac{\mathrm{\partial C}(x,t)}{\mathrm{\partial t}}=D\nabla^{2}C(x,t)-d\times C(x,t).$$

We here describe a one-dimensional (1D) system, but extending to two or three dimensions is straightforward. *C*(*x*,*t*) is the morphogen concentration at time *t* and location *x*; *D* the diffusion coefficient; and *d* the decay rate. The boundary conditions of the system are

(1b) $$\begin{array}{ll} D\frac{\mathrm{\partial C}(0,t)}{\mathrm{\partial x}}+J & =0\;,\; \end{array}$$

(1c) $$\begin{array}{ll} D\frac{\mathrm{\partial C}(L,t)}{\mathrm{\partial x}} & =0\;,\; \end{array}$$

which states that at *x*=0 there is a source of morphogen responsible for a morphogen influx, *J*, while at *x*=*L* (the length of the system) the morphogen cannot leave the system. The steady-state distribution of the morphogen concentration over space is

(2) $$C(x)=\frac{\mathrm{J\lambda}}{D\left(1-e^{-L/\lambda }\right)}e^{-x/\lambda }\;,$$

where $\lambda =\sqrt{D/d}$ is the characteristic length discussed above. Note, that when the size of the system is much larger than the characteristic length, this can be approximated as

(3) $$C(x)=C_{0}e^{-x/\lambda }\;,$$

where $C_{0}=\mathrm{J\lambda}/D=J/\sqrt{\mathrm{Dd}}$.

The fact that the measured morphogen gradient profiles in *Drosophila* strikingly follow such an exponential distribution has supported the idea that source-decay-related processes, similar to the one captured by Eq. 1, underlie the formation of these gradients [71,77,78].

#### Implications of the source-decay mechanism for the root: mathematical considerations

**Figure 3:**
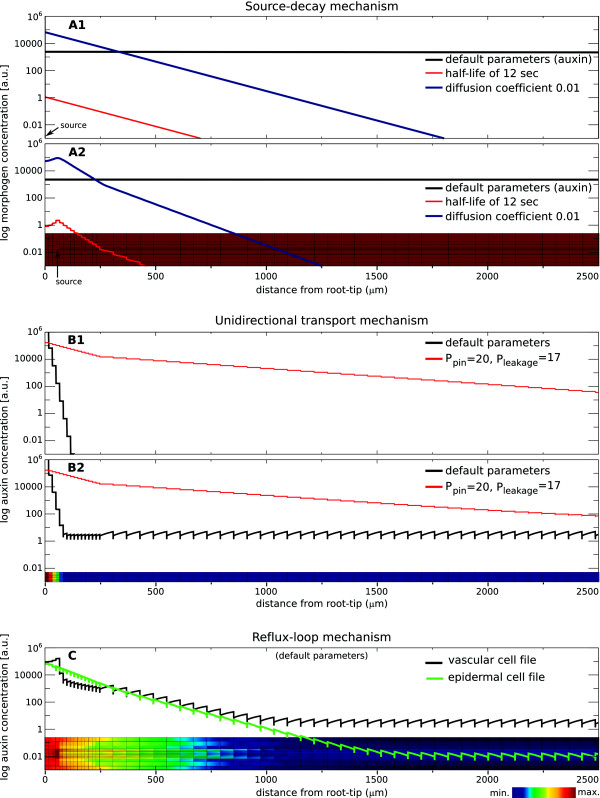
**Contrasting the steady-state morphogen gradients.** (**A**): Source-decay mechanism. (**A1**) Mathematical solutions. Default parameters for auxin (a high diffusion coefficient and a slow decay rate) result in a very shallow gradient (black). Either very fast decay (red) or slow diffusion (blue) is required to obtain a gradient with a reasonable slope and characteristic length. (**A2**) Simulations using a realistic tissue layout confirm the mathematically derived profiles: using the default parameters, the gradient is very flat (black line and inset). Only high decay (red) or low diffusion (blue) result in a reasonable gradient; while the slope is the same, the amplitude is very different. (**B**): Unidirectional transport mechanism. (**B1**) Mathematical solutions. For biophysically reasonable permeability values, an extremely steep gradient forms (black). To obtain a reasonable characteristic length, the contribution of PINs to the auxin efflux permeability has to be greatly reduced (red). (**B2**) Computer simulations on the vascular tissue layout yield similar results as the mathematically derived ones. (**C**): Reflux-loop mechanism. When using the full root layout, a reasonable gradient forms at biophysically realistic parameters. Black and green lines represent longitudinal cross-sections through a vascular and epidermal cell file, respectively. Default parameters are defined in Figure 1.

Many studies have proposed localised auxin production and/or regulated decay as an important pattern generating mechanism for the sharp auxin distributions within the root [79,80]. Utilising the equations derived above and the specific parameters for auxin allows us to quantify the extent that this mechanism could indeed be relevant. We do so for a 1D mathematically idealised root in which localised auxin production occurs at the apical end. Due to the very high diffusion coefficient of auxin and its low decay rate, the slope of the established morphogen gradient is extremely shallow (Figure 3A1). Its characteristic length, given by $\lambda =\sqrt{\frac{D}{d}}$, is around 2.4×1^04^*μ*m. Consequently, a variation of 37% is expected over a tissue length of 2.4 cm. Such a characteristic length is far too large to convey positional information to the root, because concentrations would vary only 4% over the most distal 1 mm of the root tip, where differentiation into stem cell niche, MZ, EZ and DZ take place [76]. This reveals how establishing an auxin gradient through diffusion and decay only is extremely unlikely.

However, an important question is to what extent a molecule with different kinetics (a hypothetical morphogen ‘X’) could establish a biologically reasonable gradient through the source-decay mechanism. To answer this implies ‘back-engineering’ a gradient with a realistic characteristic length of around 100 *μ*m. Such a characteristic length requires a high decay rate of around *d*≃0.06 ^*s*−1^(corresponding to a half-life of 12 sec), to compensate for the fast diffusion, or alternatively, a reduced diffusion coefficient of 0.01 *μ*m^2^/s, to compensate for the slow decay (Figure 3A1). Alternatively, instead of changing decay or diffusion independently, these two parameters could be adjusted simultaneously.

However, any modification of the parameters required to obtain the correct slope brings forth major implications. When modifying *d*, the maximum is affected dramatically. As shown in Eq. 3$C_{0}=J/\sqrt{\mathrm{Dd}}$, which implies that the modification of *d* causes a strong 250-fold decrease of the maximum. When instead *D* is modified, the time-scale of the spatial coupling becomes 6×1^04^times slower, with strong implications as well: in such a setting the morphogen would spread out very slowly (8.5 *μ*m/h instead of the 1 cm/h measured for auxin [81]). Additionally, the mean distance that a morphogen travels before decaying is equally reduced under any combination of changes in diffusion and decay. This conserved reduction is captured by the relationship (in 1D) between the morphogen’s root mean square displacement before breakdown ($\sqrt{\bar{x^{2}}}$) and the diffusion coefficient and decay,

(4) $$\sqrt{\bar{x^{2}}}=\sqrt{\frac{2D}{d}}=\sqrt{2}\lambda \;.$$

The distance molecules are typically able to move is directly related to the characteristic length, which means that in the source-decay mechanism fixing the characteristic length implies fixing the length of communication.

Thus, to assume an alternative morphogen for auxin implies that on average a pulse is expected to travel no further than 140 *μ*m through the plant. This is very different to the observations showing that a pulse of auxin can travel large distances [51]. It would exclude the morphogen forming the positional informative gradient to function simultaneously as a signalling molecule which integrates information regarding the whole plant system, as auxin is known to do [82].

#### Implications of the source-decay mechanism for the root: a computational analysis

The idealised 1D mathematical description of a source-decay mechanism clearly ignores a number of aspects of the root related to its cellular structure. To determine if the insights from the mathematical analysis still hold when the cellular structure and the organisation of the tissue structure are explicitly taken into account, we performed computer simulations of the *Arabidopsis* root (Figure 1). We use typical cell sizes and PM permeabilities for auxin [see [53,58]], as well as the 15-fold reduction in auxin diffusion in the cell wall [69]. Auxin fluxes over the PM are given by

(5) $$\;\vec{F}=\{\begin{array}{ll} (P_{\text{pin}}\hat{n})C_{\text{in}}-(P_{\text{aux}}\hat{n})C_{\text{out}} & \text{if PINs are expressed},\\ (P_{\text{bg}}\hat{n})C_{\text{in}}-(P_{\text{aux}}\hat{n})C_{\text{out}} & \text{if only background}\\ \;\text{efflux takes place,} \end{array}$$

where $\hat{n}$ is the outward-directed unit vector, perpendicular to the PM; _*Ppin*_represents the efflux permeability over the membrane in the presence of PIN expression; _*Pbg*_ represents the much lower background efflux permeability through the membrane itself in the absence of enhanced PIN-mediated transport; and _*Paux*_ represents the influx permeability. The latter is in the same order as _*Ppin*_, because of the higher passive influx rates due to chemiosmotic considerations and the action of AUX/LAX auxin influx transporters. _*Cin*_ represents the auxin concentration in the cytosol at the PM; and _*Cout*_represents the auxin concentration in the cell wall immediately adjacent to the PM. In the model, we consider the source of auxin to be localised at the point of the maximum, the ‘QC’.

To exclude the role of polar transport (in order to analyse purely the source-decay mechanism), we assume high PIN expression along all sides of each cell, unlike what is depicted in Figure 1C.

In the simulation a gradient is established which is indistinguishable from the mathematically derived 1D gradient (compare black lines depicting equilibrium profiles in Figure 3A2 and A1). Whether or not the cellular structures are taken into account, using this mechanism, auxin would generate a too shallow gradient for positional information. Also the simulations that consider alternative morphogens, with increased decay (*d*=0.06 s^−1^) or reduced diffusion (D = 0.01*μ*m^2^/s) present very comparable equilibrium profiles (Figure 3A2) as predicted from Eq. 3 (Figure 3A1). Thus, details introduced by the cellular structure do not play a role within this mechanism.

#### Unidirectional transport mechanism

It has long been known that auxin does not only spread through the plant through passive diffusion, but that directed auxin transport is involved [81,83-86]. Model studies in the early eighties have shown that a unidirectional transport mechanism could underlie the establishment of auxin maxima [59,68]. These models predicted the existence of polarly localised auxin efflux facilitators, which only much later were experimentally found, i.e. the family of PIN proteins [87].

**Figure 4:**
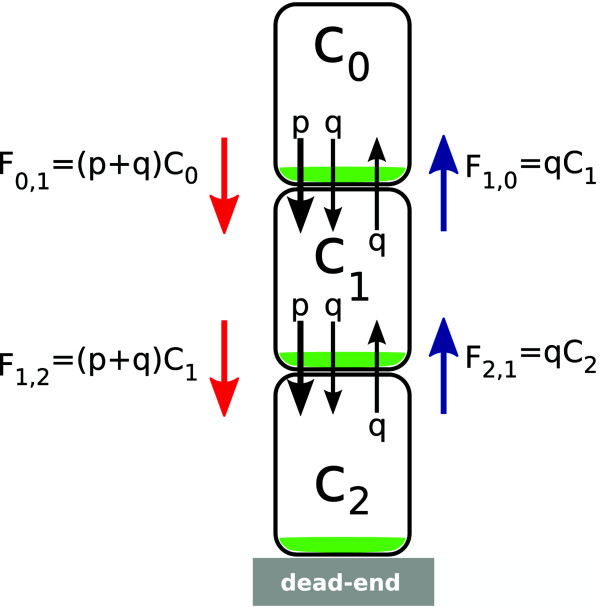
**Unidirectional transport mechanism.** Schematic figure to guide derivation of established gradient.

Unidirectional transport is not only able to generate a maximum, but also a morphogen gradient. The most direct mathematical way to derive the effects of unidirectional transport is to consider a single cell file containing *n* cells that transport auxin directly into their neighbouring cells in the downward direction (from cell *n*=0 to cell *n*=*N*) (Figure 4 shows a simplified example). The role of intracellular diffusion and decay is ignored, and consequently only one concentration, _*Cn*_, has to be considered for each cell.

In spirit of Mitchison’s derivation, the equilibrium distribution can be easily derived and generalised from a simple example of only three cells as shown in Figure 4. The first cell, *n*=0, is kept at a constant auxin concentration _*C*0_. Auxin transport is indicated by the red and blue flux arrows in the figure. In the absence of localised polar efflux carriers, auxin fluxes out of the cells with permeability rate *q*. When polar efflux carriers are present, indicated in green in Figure 4, auxin efflux is augmented with *p*, such that the total permeability rate becomes (*p* + *q*). The auxin flux from cell 0 to cell 1 is _*F*0,1_=(*p* + *q*)_*C*0_. The auxin flux in the opposing direction, i.e. from cell 1 to cell 0 is _*F*1,0_=*q*_*C*1_. Given that at equilibrium _*F*0,1_=_*F*1,0_, the concentration _*C*1_ at equilibrium can be expressed in terms of the concentration at _*C*0_, through

(6) $$C_{1}=\frac{p+q}{q}C_{0}\;.$$

Similarly, if we equalise the fluxes between cell 1 and 2, we find that $C_{2}=\left((p+q)/q\right)C_{1}$. Using the expression for _*C*1_above, this becomes

(7) $$C_{2}=\frac{{\left(p+q\right)}^{2}}{q^{2}}C_{0}\;.$$

In a general form, the dynamic changes in morphogen concentration in cell *n* can be described mathematically as

(8a) $$\frac{dC_{n}}{\mathrm{dt}}=(p+q)C_{n-1}+qC_{n+1}-(p+2q)C_{n}\;,$$

with boundary conditions

(8b) $$\begin{array}{ll} \frac{dC_{0}}{\mathrm{dt}} & =0\;,\; \end{array}$$

(8c) $$\begin{array}{ll} \frac{dC_{N}}{\mathrm{dt}} & =(p+q)C_{N-1}-qC_{N}\;.\; \end{array}$$

Through an iterative process as described above, the equilibrium distribution for the general form yields

(9) $$C_{n}={\left(\frac{p+q}{q}\right)}^{n}C_{0}=C_{0}e^{\frac{n}{\lambda }}\;,$$

where _*C*n_ is the concentration in the *n*-th cell along the cell file, and $\lambda =1/\log\left(\left(p+q\right)/q\right)$, expressed in cell lengths. The equilibrium distribution presents an exponential increase in auxin along the cell file. Importantly, in contrast to the source-decay mechanism, here the source of the morphogen is located at the far end of the gradient. The generation of such a profile can be referred to as occurring due to ‘heaping up’ of auxin, given that a constant active transport up the gradient towards a ‘dead end’ underlies the mechanism. The slope of the gradient greatly depends on the parameter values of the specific downward-directed PIN permeability rate *p* and on the leakage *q* that acts equally in both directions^b^. The location of the maximum is determined by the end of the cell file in which further transport is blocked, causing auxin to heap up at that location to the highest levels.

#### Implications of the unidirectional transport mechanism for the root: mathematical considerations

We now ask what type of gradient would be expected when considering a realistic vascular cell file with unidirectional transport. Given that the downward transport takes place through the vascular tissue, the derivation shown above resembles a vascular cell file, for which basally-oriented polar PIN localisation has been experimentally verified [51,74,87]. As discussed above, background permeability, equal along the entire PM of the cell, is _*Pbg*_=1 *μ*m/s, while within the vascular cells additional permeability due to PINs localised along the basal PM (as depicted in Figure 1C), gives rise to an increased permeability along the basal PM of _*Ppin*_=20 *μ*m/s. Translated back into the parameters of Eq. 8, this results in *p*=19 *μ*m/s; *q*=1 *μ*m/s. Using Eq. 9, it follows that concentrations would drop 20-fold with each cell (_*Cn*_/_*Cn*−1_=20), the characteristic length *λ* being $1/\log\left(\left(p+q\right)/q\right)=0.33$ cell length, or ≃5 *μ*m. Thus, within such a cell file, auxin concentrations drop more than 19 orders of magnitude over the first 15 cells from the maximum. This limits the functionality of the auxin gradient to only very few cells close to the maximum, as can be seen in Figure 3B1 (black line), which plots the mathematically predicted auxin gradient (Eq. 9), using a vascular cell template to correct for cell lengths. The value of _*C*0_ is determined by assuming a total amount of auxin within the vascular bundle equal that used in the source-decay mechanism. Again, this has strong consequences for the positional information over the root, but in an opposite way as was observed for the source-decay mechanism: here the gradient is far too steep, while previously it was too shallow.

The above reasoning depends on quantitative aspects related to the specific PIN localisation and permeability rates. However, here again we can ask what would be needed for this mechanism to form a reasonable gradient, with a characteristic length of around 100 *μ*m, or 6–7 cell lengths. Permeability parameters can be ‘back-engineered’, revealing that background permeability has to be dramatically increased (*p*=3 *μ*m/s; *q*=17 *μ*m/s). Indeed, this leads to a reasonable equilibrium slope and maximum (Figure 3B1, red line).

These modifications in permeability rates clash in several ways to experimental observations and requirements on auxin transport. First, at the required parameter settings, polar PIN expression only causes a small increase in permeability, less than 18% compared to the background permeability. This contradicts the chemiosmotic properties of auxin described above, which states that without auxin efflux facilitators the permeability of the PM for cytosolic, anionic auxin is very low [59,72,88], and that the contribution of PINs to auxin efflux plays a substantial and predominant role, compared to other known or possibly undiscovered efflux carriers, given that *pin* mutants strongly reduce vascular auxin transport [87]. Secondly, experiments show a fast and directed pulse propagation through plant tissue [51,81]. At the required parameter settings derived above, pulse propagation becomes an order of magnitude slower compared to the default setting [as can be calculated using the second equation in [59]] and much slower than the known typical transport rate [81]; additionally, the pulse would rapidly become ‘smeared’ out rather than being transported more or less as a whole [51]. Thirdly, in this mechanism the maximum is being formed at an effective ‘dead end’. However, it has been shown that QC cells at the maximum strongly express PINs, without any indication of transport inhibition [51]. Thus, this mechanism fails to predict a maximum at PIN-expressing cells, as is the case for the QC. To conclude, although it can explain the rapid formation of a strong auxin maximum, due to these three issues it is hard to reconcile how the unidirectional transport mechanism could give rise to a correctly positioned informative auxin gradient within a tissue that still reveals ‘root-like’ properties. Nevertheless, given only these spatial considerations, one could still imagine that a root with different transport features could have been evolved using such a mechanism. We will see below that more important drawbacks emerge when considering the temporal dynamics of gradient establishment.

#### Implications of the unidirectional transport mechanism for the root: a computational analysis

For the ease of analysis, the 1D mathematical description of unidirectional transport used a number of simplifications (such as not treating intracellular diffusion, ignoring the cell wall, and neglecting auxin degradation). We therefore ask whether better descriptions of auxin transport as well as the cellular embedding within the root layout affect the mathematical reasoning given above. We explore this by simulations of unidirectional transport within a realistic vascular tissue layout, which only considers the vascular and pericycle cell files and the QC, using zero flux boundaries. The simulations consider cell walls, multiple cell files and auxin diffusion in the cells and within the cell wall, using realistic diffusion coefficients and decay rates. Moreover, both PIN-mediated polar efflux as well as the influx over the PM are explicitly described, using Eq. 5. The simulation of the unidirectional transport in the vascular tissue indeed generates a maximum at the ‘dead end’, i.e. the QC, as well as a very sharp auxin gradient, matching very closely the gradient predicted by the mathematical caricature (compare Figure 3B2 with Figure 3B1, black lines), demonstrating that neither the intracellular diffusion nor moderate decay rates impact the expected profiles for the unidirectional transport mechanism. After five cells auxin has dropped to extremely low levels, confirming the short characteristic length calculated above from the idealised situation. The main new feature revealed through the simulation is that due to auxin entering and decaying in the root, the distal gradient is connected to a proximal influx-driven, almost flat profile. The fact that the magnitude of the maximum and slope of the gradient match between simulation and idealised root, justifies the simplifications used for the mathematical analysis.

To back-engineer a biologically relevant gradient within the *in silico* tissue, we introduced apically localised PINs with a permeability rate of 85% of the basally localised PINs. Again, the profile very closely matches the mathematically derived one (Compare Figure 3B2 with Figure 3B1, red lines). The influx-driven part of the profile reaches much higher concentrations, because downward transport is effectively an order of magnitude slower. Note that the resulting slope is constant when expressed per cell (as predicted from the mathematical analysis), and therefore becomes much more shallow within the EZ composed of longer cells, when expressed in length (*μ*m). Thus, the computer simulations reconfirm the inconsistency between the necessary requirements in the model and experimental data, as described earlier.

The contrast is large between the source-decay mechanism and the unidirectional transport mechanism. Although in both cases a similar root layout was used and similar kinetic parameters and cell sizes were considered, we find that in the first case the gradient is determined by diffusion and decay, while those parameters do not play a role in the second case. Likewise, transport rates determine the gradient in the second case, which do not play a role in the first. In both cases the location of the maximum has to be determined at forehand, either by setting the source of auxin at the location of the QC or by forming at this location a dead end. Also, both mechanisms present drawbacks when parameters are modified to obtain a realistic characteristic length. Finally, neither mechanism generates a functional gradient for biophysically known parameter values of auxin.

#### The reflux-loop mechanism

We have previously shown, through combined modelling and experimentation, that the mechanism by which a gradient is being formed in the *Arabidopsis* root is a different one than those mechanisms previously discussed [53]. The ‘minimal root’, depicted in Figure 1B,C captures the essential properties of this tissue. The core differences with the unidirectional transport mechanism are located in the external cell files: upward transport due to apically localised PINs and lateral transport due to PINs localised to the inner lateral walls of the cells (facing the midline of the root).

Because of the overall complexity of the layout and multilevel interactions between cell and tissue properties, in this case, a mathematical caricature of this mechanism is not easily derivable. Instead we immediately move to the full computational analysis. Figure 3C shows the profile to a vascular cell file and an epidermal cell file when the simulation is run for the default parameter settings. The resulting equilibrium gradient presents a functional slope and a maximum positioned at the QC.

Auxin is transported downwards through the vascular tissue, reaching the most distal cells, the root cap. The root cap connects the basal-directed auxin flow of the central vascular region to the apical-directed flow of the external cell files, through apolar PIN localisation in the columella cells. Laterally localised PINs promote the lateral auxin flux from the external cell files back into the vascular bundle, closing a reflux-loop. The reflux-loop causes the formation of an exponentially increasing auxin gradient that spans the entire MZ and part of the EZ. The observation that the reflux-loop mechanism generates an exponential gradient can be understood as follows. Due to the laterally localised PINs, at any vertical position a small fraction of the auxin within the external cell files fluxes laterally back into the vascular tissue, while a large fraction continues its flux upwards. In the limiting case that (i) the fraction entering the vascular tissue at any vertical position is constant, and (ii) the flux within the vascular tissue is solely downwards, the concentration drop within the external cell files can be captured by *dC*/*dy*=−*αC*, where *α*describes the flux back into the vasculature. This gives rise to an exponential profile. The profile within the vasculature has to present a similar exponential profile, given that the increase in vascular concentration is due to the same lateral flux. The exponential shape can thus be attributed to the lateral flux, with the strength of the lateral flux determining the steepness of the auxin slope. The highest concentrations form within those cells that lie at the interface of the downward flow through the vascular tissue and the upward flow through the external cell files, i.e. within the QC cells. Similarly to what occurred in the unidirectional transport mechanism, the distal exponential part of the auxin distribution is connected with a flat, influx-driven profile within the proximal region, forming an ‘elbow’ at their junction (Figure 3C). The flat profile extends from the distal EZ proximally, into the DZ.

In short, the essential requirement for the reflux-loop mechanism is the existence of a lateral flux linking two oppositely directed fluxes. It results in an exponential increase in auxin towards the distal end, both in the vasculature and in the external cell files. The precise location of the increased inwards permeability is not important for the mechanism; although strong lateral PIN expression within the endodermal cell file leads to the highest auxin levels within the QC, the mechanism also functions when lateral PIN expression is mainly or solely restricted to the cortex or epidermis (results not shown).

Contrasting this mechanism with the previous, brings forth a number of important differences. Firstly, the correct distribution is found for the known parameter values for auxin transport. Secondly the QC is not a pre-specified source of auxin, as in the source-decay mechanism, and accumulation at the QC occurs within a realistic root layout, in contrast to the requirements on tissue structure imposed by the unidirectional transport mechanism. In fact, it is not that the reflux-loop functions despite the root cap, but rather that the root cap is essential for generating the maximum at the QC. Thirdly, whereas in the previous mechanisms the fluxes through the tissue nearly disappear when the auxin profile reaches its equilibrium, within the reflux-loop they remain very high, also at steady state (Figure 1). The highest throughput is found at the maximum, the QC, in sharp contrast with the unidirectional transport mechanism, where the maximum is a ‘dead end’ for auxin flow. Note that such high fluxes cause the formation of intracellular gradients that have been suggested to play a role in development [58,89]. More differences, however, become apparent when we not only look at the steady state distribution, but also take the dynamics of the gradient formation into account.

### Trade-off between spatial and temporal scales

As indicated in the introduction, to evaluate the functionality of a morphogen gradient, the temporal dynamics of the mechanism should also be analysed.

#### The establishment of a gradient

In an attempt to ‘match’ the steady state profiles of the three different mechanisms, specific requirements were derived for each mechanism, proposing hypothetical morphogens or tissues with different transport properties. We found that in the case of the source-decay mechanism, the requirements greatly restrict the expected distance a molecule would be able to travel through the tissue. Here we continue exploring the effects of the temporal dynamics, but from another angle, by looking at the transient behaviour of the morphogen distributions. How do the different pre-steady-state distributions look like and how fast do they form?

**Figure 5:**
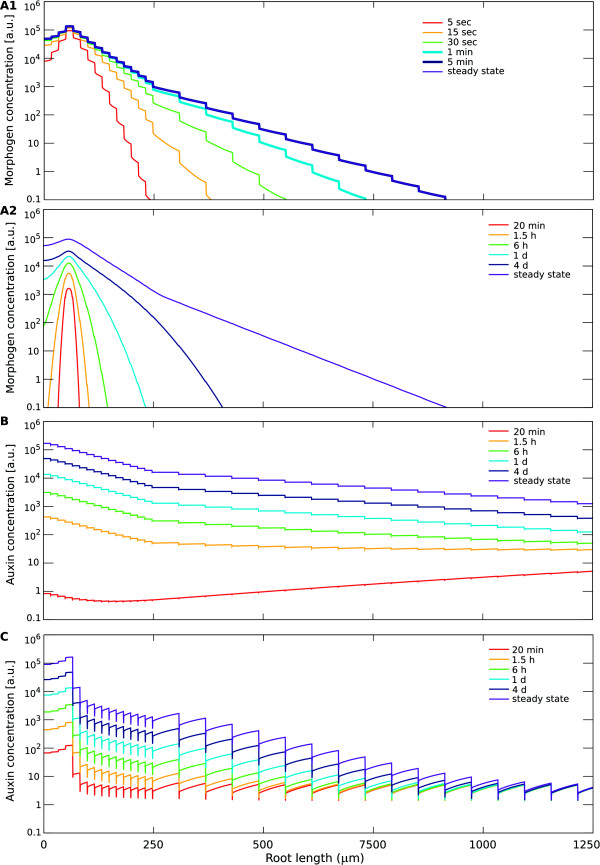
**Contrasting the time-scales for morphogen pattern formation in the different mechanisms.** Computer simulations were performed for the (**A**): source-decay; (**B**): unidirectional transport; and (**C**): reflux-loop mechanism. Simulations are done as described in Figure 3, for the parameter settings described therein that generate reasonable gradients, with one modification, which is using for the source-decay model with fast decay an equally increased production rate. At *t*=0 the tissue is free of morphogen/auxin. Graphs show morphogen profiles along a longitudinal cross-section through a vascular cell file at different time points, indicated by the colours. (**A1**) With high decay rates, the source-decay mechanism quickly reaches the exponential steady state. The required high influx rate needed for this system to acquire similar morphogen concentrations as the other models ensures the formation of the maximum after already 5 s. Within 5 min the steady state is reached. (**A2**) With slow morphogen diffusion, the source-decay mechanism presents an extremely slow progression towards the steady state pattern. Even after 4 days the maximum is still building up, and the tail of the distribution fails to span a larger tissue region. (**B**) The unidirectional transport mechanism initially develops an inverted gradient, only after 1 h concentrations at the tip become higher than elsewhere. Thereafter, the pattern remains relatively similar, while concentrations slowly rise over the whole tissue. (**C**) The reflux-loop mechanism quickly establishes an exponential profile with a characteristic slope, forming an ‘elbow’ with the proximal, flat influx-driven gradient. As time progresses, the slope of the exponential profile is conserved, while the overall absolute values increase, but only in the distal region, allowing the ‘elbow’ to shift proximally. The formation of the gradient (maximum and slope establishment) occurs on very fast time-scales, while the ‘shift’ in the slope along the tissue occurs on a much slower time-scale.

We compare the dynamics of the three mechanisms, under the biophysical parameter regimes in which they each generate the similar biologically relevant exponential steady state profile and maximum using the parameter settings of Figure 3, but with one modification: in the case of source-decay with fast decay, the source has been equally modified to keep total morphogen content the same (Figure 5).

The source-decay mechanism is generating very contrasting dynamics when either fast diffusion (and fast decay) or slow diffusion (and slow decay) are considered (Figure 5A1 and A2), even though the slope at steady state is equal for both hypothetical morphogens. While with fast decay a gradient forms within 5 minutes, in the alternative implementation with slow diffusion it takes many days. Moreover, the transient profiles are not exponential, with the tail of the distributions dropping very steeply. The very slow time-scales that accompany the formation of a spatially relevant gradient is a major shortcoming of the source-decay mechanism with slow diffusion (Figure 5A2). These issues can be overcome with high decay rates, but this requires a huge increase in morphogen production, which might be costly for the QC (Figure 5A1).

The unidirectional transport mechanism also presents relatively slow time-scales (Figure 5B), but far above that of the source-decay with slow diffusion. The maximum establishes within one hour, after which it steadily increases in magnitude. The reflux-loop (Figure 5C) forms on a very short time-scale of just minutes a high auxin maximum and a gradient. As total auxin amounts increase, due to auxin influx, the absolute values at the gradient increase while it maintains the same slope over time. The reflux-loop accounts for very different time-scales concomitantly: whereas the establishment of the auxin maximum with the characteristic slope occurs on a very fast time-scale of minutes, the gradient presents a slow spatial shift over a time-scale of days. The slower time-scale of the spatial shift that we observe in the simulations matches observed MZ extension rates that were found experimentally in growing roots [53]. Moreover, after root severing (which terminates the influx of auxin from the shoot), we can observe the retraction of the MZ over a period of many days, again closely matching the simulations with the experiments [53]. (Note that these long time-scales reflect the capacity of the reflux-loop mechanism to be able to “store” large quantities of auxin within the loop.) The short time-scale allows this mechanism to ‘keep up’ with the typical root growth, around 800 *μ*m/h [76]. We have shown this before in simulations which also implemented auxin-regulated cell divisions, cell expansion and root growth, using a modified Cellular Potts Model [see [53]]. Slow diffusion and, to a lesser extent, unidirectional transport are not able to accompany such a dynamic tissue growth, which is again an important drawback of these mechanisms for plant organ development, i.e. for systems that grow so rapidly and become so large. A source-decay mechanism with fast decay precludes such developmental regulation on such a long time-scale.

Taken together, the combined analysis on the requirements for a correct spatial patterning and its implications for the dynamics of the morphogen gradient is fundamental in determining the functionality of a gradient-forming mechanism. Simply put, although it is important to generate a characteristic length of 100 *μ*m, this will be of little use to the developing root, if the pattern is only established after many days. Additionally, the long time-scale generated by the reflux-loop can be correlated with the experimental observations on the dynamics of the zonation [as the root grows, the MZ expands, [76]]. Thus, the dynamics of the reflux-loop mechanism and its role in the root zonation dynamics, indicates that the transient profiles of the gradient can be as important – and arguably even more so – than the steady state profile.

#### Ablation of the morphogen maximum

**Figure 6:**
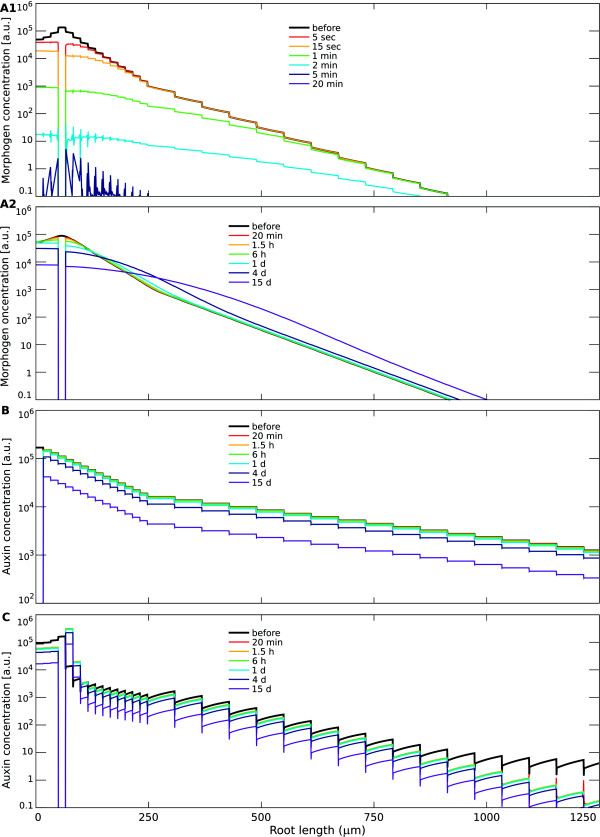
**Contrasting the time-scales for maximum reestablishment in the different mechanisms.** Computer simulations were performed for the (**A**): source-decay; (**B**): unidirectional transport; and (**C**): reflux-loop mechanism. Simulations are done as described in Figure 5. The simulations are initiated with the steady state morphogen distribution. At *t*=0 the morphogen influx ceases and the QC cells, containing the maximum, are removed. Graphs show morphogen profiles along a longitudinal cross-section through a vascular cell file at different time points, indicated by the colours. (**A1**) With high decay rates, the source-decay mechanism presents a dramatic disappearance of the morphogen in the root tip after ablation of the source: all morphogen disappears within 5 min. (**A2**) In contrast, with slow morphogen diffusion, the source-decay mechanism maintains a maximum over long time-scales (15 days). However, especially in in the vicinity of the removed source, the slope fades out within a few hours. (**B**) The gradient of the unidirectional transport mechanism remains unaffected by the disappearance of the maximum. On a short time-scale all cells maintain the same concentration. Due to the auxin decay and interruption of the influx, auxin values decrease homogeneously over the whole tissue on a longer time-scale, such that the new ‘maximum’ continues to diminish in magnitude. (**C**) In the reflux-loop mechanism there is a quick reestablishment of an auxin maximum, with a small region just above the removed maximum presenting a fold increase in auxin concentrations. On a longer time-scale the overall concentrations decrease due to auxin decay and lack of influx, bringing down the absolute level of the maximum.

Another way to contemplate the impact of the mechanism is by observing the morphogen dynamics when challenged by external interferences. For example, experiments in which the QC cells has been ablated, verified the reappearance of an auxin maximum just above the ablated tissue, leading to the reestablishment of a functional stem cell niche and root patterning on remarkably short biological time-scales [52]. To test further the role of auxin influx to this auxin maximum reappearance, we repeated these experiments using an amputated root (shoot removed), and again verified the similar robust and fast dynamics of auxin reestablishment [53]. In Figure 6 such an interference (i.e. both blocking any auxin entry from the shoot and removal of the QC) is simulated for all mechanisms (for the same parameter settings as Figure 5). Note that the *in silico* ablation is implemented by fully obstructing auxin flow into the area previously occupied by the ablated cells (i.e. computationally the cells are “ablated” by changing the region they occupy into no-flux boundary conditions). This implementation completely ignores the cellular responses which the obstruction and the changing auxin distribution might cause on a longer time-scale (either directly or indirectly), such as changes in polarity, PIN-expression levels or modifications in production and decay rates, all of which are not treated here.

For the source-decay mechanism, the elimination of the QC implies the absence of the source, and hence, the impossibility not only to reestablish a maximum, but also to maintain the gradient (Figure 6A1). Most dramatically, when decay is fast, all morphogen disappears from the root within minutes (Figure 6A2), showing that what was optimal in the previous section now works out the worst. The system driven by the unidirectional transport mechanism cannot quickly increase auxin in the neighbouring cells after ablation (Figure 6B), as it requires influx of auxin from the shoot over a long time period to re-accumulate auxin above the ablated cells.

Interestingly, only the reflux-loop mechanism presents a region in which concentrations *increase* after root cut and QC ablation. This region, next to the original QC and coinciding with the redifferentiation of the new QC, presents a fold increased auxin concentrations within 20 min. This is due to the reflux-loop generating constantly high fluxes, causing a rapid replenishment of the cells just above the removed QC. In contrast, the other mechanisms, independent of parameter choice, only present decreasing morphogen levels in every single cell. Thereby, only the reflux-loop enables the root to store effectively auxin in the apical region, in a form which generates a great degree of autonomy from fluctuations in the influx and damage to the stem cells at the root tip.

#### Auxin be nimble, auxin be quick

When comparing properties of plant development and measurements of auxin kinetics to animal development and their involved morphogen gradients two obviously contrasting features become evident. The first is the difference in space-scales and the second is the size of the molecule being utilised as the morphogen. In the above analysis we have shown that these two points are in fact related.

Plant development not only occurs during embryogenesis, but continues over the whole life cycle of the plant, and thus, morphogen gradients are relevant within the context of very large tissues. Moreover, a plant morphogen not only needs to establish robust positional information, but also guide tropism by quickly changing and transferring information over long distances.

Typically, known morphogen gradients, such as Wg in *Drosophila,* do not face comparable challenges, because the scale of the embryo is much smaller, such that the region over which the gradient acts and the distance over which it has to be transported is much more limited. Given that the morphogens in such systems tend to be proteins, with a much higher molecular mass than auxin, they have much lower diffusion coefficients which allows for concentrations to vary significantly over the relatively small distance within the embryo.

The reflux-loop is able to satisfy both long-distance communication and robust positional information: by utilising such a fast molecule as auxin, issues regarding long-distance transport and communication can be solved; by dynamically maintaining high fluxes, different time-scales can be accounted for simultaneously; and through lateral fluxes, robust, steep gradients can be formed. However, what are its dependencies to biophysical parameters, and how can the plant fine-tune its gradient through the reflux-loop mechanism?

### Jumping over biophysical limitations

**Figure 7:**
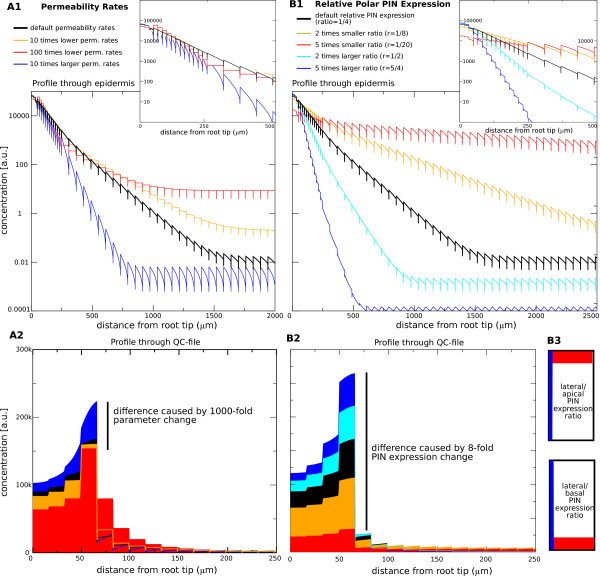
**Robustness of the gradient.** (**A**): Robustness of the gradient within the reflux-loop mechanism, shown through a 1000-fold change in the permeability values. To facilitate the analysis on the effect of changes in the permeability rate, the net influx from the shoot is kept constant (basically assuming that the auxin biosynthesis in the whole plant is independent of the transport rates). (**A1**): Logarithmic auxin profiles along a longitudinal cross-section through a epidermal cell file. The first 2000 *μ*m are shown. The inset shows a zoom-in close to the maximum, illustrating a marginal change in slope. (**A2**): Linear auxin profile along a vascular cell file, illustrating the marginal change in the maximum value. (**B**): In contrast, only a 2-fold change in the ratio between the apical or basal and the lateral permeability rate leads to dramatic changes in both the maximum and slope of the gradient. Profiles are as in (A), except that the first 2500 *μ*m are shown. The differences in apical/basal (red) versus lateral (blue) PIN expression in the epidermal and cortex cells, which determine the slope of the gradient, are schematically shown in (**B3**).

Often the concept of morphogen gradients is criticised for its biophysical limitations, which would render it inflexible (diffusion coefficients can only be modified to a certain extent), and would limit its applicability to a typical spatial range. Moreover, the established gradient would depend heavily on the noise from the source or influx [90]. To start with the first point, changes in localised production or decay within the reflux-loop mechanism does not at all affect the established pattern (as is the case within the source-decay mechanism), as long as the diffusion and decay are within the wide, biologically relevant parameter regime. Such a regime is obtained when considering reasonable auxin transport rates and mean travel distances as found in experiments. When these conditions are met, the specific location of production and/or decay is effectively ‘invisible’ for the auxin pattern that is established [53]. However, higher production and/or higher decay can change the overall level of the auxin gradient, and regions of regulated auxin biosynthesis and catabolism could be important to modulate the reflux-loops distributions [91]. Moreover, also changes in permeability rates or cell sizes do not affect the established pattern (as is the case within the unidirectional transport mechanism). Within the reflux-loop 1000-fold variations in permeability rates only marginally changes the slope and maximum (Figure 7A). A similar robustness occurs when the background permeability is fixed while the PIN-mediated permeability is varied 100-fold (results not shown). Thus, none of the biophysical parameters have a strong effect on the established auxin maximum and gradient. Since the slope is not determined by the biophysical parameters, a gradient can be established of any slope, and due to the continuously high fluxes communication is still possible over a very wide range of spatial scales. And, given the reflux-loop capacitance to ‘store’ auxin within the distal region, it is very robust towards fluctuations in influx.

In contrast to being dependent on biophysical parameters, the auxin gradient generated through the reflux-loop mechanism can be carefully controlled on the *cellular level*, through the tuning and modification of the ratio in the lateral to apical PIN expression of the ground tissue cells (Figure 7B). Very small variations in this ratio have a huge impact on the slope and maximum. This, however, is a question of regulation of cell polarity, bypassing altogether the standard biophysical limitations. These results emphasize again the essence of the reflux-loop mechanism, in which the ratio between the effective lateral and upward flux is what most determines and impacts the characteristic length of the resultant exponential gradient. We here focused on the impact of specific parameter choices on the profile, such as modifications of the slope due to the magnitude of the lateral flux as determined by PIN-related permeabilities. Note, however, that topological changes in the tissue layout, such as modifications in the number of root files that express lateral PINs or changes in the specific file(s) which do so (for example, endodermis vs. epidermis), will also lead to changes in the effective lateral flux in relation to the upward flux, which in turn causes alterations in the characteristic length of the exponential gradient (data not shown).

Together this reveals the striking robustness of the reflux-loop mechanism with respect to variations in PIN permeability or any other biophysical parameter. Thus, the reflux-loop not only ensures that the slope of the gradient becomes much steeper than is possible with a source-decay mechanism, relying on a fast diffusing morphogen such as auxin, it also loosens the strict dependency on absolute permeability and leakage rates by using topological tissue properties and cellular-based PIN expression, instead of specific kinetic constants, to regulate the slope.

## Conclusions

While evidence is pouring in on the exceptional role of auxin in establishing spatial patterns, there has been some reluctance in designating the auxin concentrations along the root tip a ‘morphogen gradient’. Three factors forge this reluctance. The first problem is that often a narrow definition is used for the manner in which a morphogen gradient ought to be generated. The intense research and advances in *Drosophila* and *Xenopus* development, which helped conceptualise the role of morphogen gradients, has also contributed to the notion that it, per definition, has its maximum at the location of morphogen production, establishing a gradient through processes linked to diffusion and decay. In contrast, we here compared several gradient-generating mechanisms to determine their ability of conferring biologically robust and clear information through the generated gradients. We found that the source-decay mechanism presents severe limitations in the context of the growing root, thereby generating a strong case why morphogen gradients ought not to be defined like this. Indeed, Wolpert, when formulating the idea of positional information in the canonical article [2], pointed out that “one needs to specify the mechanism generating a gradient”.

The second problem concerns the output. Traditionally, discussions about morphogen gradients focus heavily on fate changes, while in plant development ‘guided differentiation’ much better describes the action of auxin gradients. As a consequence of cell divisions and the growth of the root tip, cells effectively ‘move through’ the auxin gradient, which simply follows the growth of the root tip. The changing auxin concentrations are then instructive to change the cellular behaviour from MZ-like to EZ-like, and possibly, to DZ-like, while maintaining the stem cell niche.

The third problem is whether auxin has a direct effect (i.e. on the cellular level) on the cell differentiation. Could instead some of the alternative mechanisms for cell fate changes and cell differentiation as discussed in the introduction be involved? This is a complex issue to verify. First, the modelling shows that due to the high throughput it becomes experimentally challenging to change locally the auxin content of a specific cell without also dramatically modifying its transport properties, with all its possible consequences. Second, verification through the manipulation of auxin effectors is also not foolproof, as there is evidence that the downstream factors influence transport and hence the distribution of the upstream gradient that controls their expression [56]. However, the direct action of an auxin gradient can still be considered the most parsimonious explanation for all experimental data up to date [7].

Given the continuous development of plants, structured spatial regions that span very large distances (when compared to animal systems) have to be established, while precise cell-to-cell variations maintained. Also, the manner in which plants integrate environmental information into developmental outputs, adds extra requirements for the gradient-generating mechanism. This is because both the scale and the plasticity of plants calls for fast information transfer. Realising this, it is not surprising that auxin, a small, fast diffusing molecule, and directed transport underlie many aspects of plant development. In this respect, we see that both a source-decay mechanism as well as a unidirectional transport mechanism fail to bring forth concomitantly a stable robust pattern that spans a relevant tissue segment, while also being dynamic enough to alter and transfer information over large distances within small time intervals. Both properties simultaneously co-exist in the reflux-loop mechanism.

The insights gained from our mathematical analysis and computer simulations regarding the source-decay mechanism might have implications for the understanding of plant gametogenesis. Recent findings point towards an auxin gradient, but polar auxin transport has not yet been observed in this context [92]. In our study we have shown that when there is no polar transport, a gradient that has an informative characteristic length will drop rapidly to very low absolute concentration levels (see Figure 3 red lines), and therefore would only be effective over a small spatial distance. This is a consequence of the very fast diffusion of the molecule having to be compensated by a fast decay rate. However, given that gametogenesis – unlike most of plant development – occurs on restricted small spatial scales (the developing embryo sac grows from 30 to ∼100 *μ*m), fast auxin decay rates would suffice to establish a source-decay-driven gradient. The discussed drawbacks that high decay rates evoke (such that an auxin pulse cannot be transmitted over larger distances), would not hinder a system of such a small size. Moreover, unidirectional transport efficiently establishes patterns on small scales, that is, it quickly leads to large concentration differences over just a few cells – a property which might also be essential during the first stages of plant embryogenesis. In this manner, it is possible to imagine, that during the whole life-cycle of a developing plant, various combinations of mechanisms can be used in an overlapping manner, with some more dominant than others during specific moments of development. The regulatory mechanisms that could allow a gradient-forming mechanism to build upon a previous one is of course open for speculation. One possibility could be that once a gradient is established through a source-decay-driven mechanism, this could be picked up by intracellular polarity mechanisms, triggering the formation of rows of cells with orientated polarities and coordinated polar transport. This, in turn, would give rise to a unidirectional transport mechanism that could ensure precise cell differentiation due to the extreme concentration differences it could generate. Through the formation of new cell files the unidirectional transport mechanism could then slowly be transformed into a reflux-loop mechanism, thereby acquiring cell-polarity-based control over the slope and spatial range of the gradient while conserving the rapid time-scale for communication. Such a hypothetical scenario, however, requires not only regulation of both cell and tissue polarity, but also the progressive establishment of cell identity and differentiation. Hence, many fundamental questions regarding this problem remain to be answered.

Having identified the key requirements for the reflux-loop mechanism to generate gradients within the root also allows us to treat the mechanism in a more abstract form, to serve as a powerful search-image for other phenomena of pattern formation, not only in plant development (such as in lateral root formation [58]), but also in animal systems (it could for example play a role in the functioning of kidneys, see [93]). Even outside the realm of development the “reflux-loop mechanism” might be functional in biological systems. For example, the mechanism proposed in Baaske [94] for generating sufficiently high chemical concentrations for prebiotic biochemistry to take off could be related to the mechanism presented here.

Our “Gedanken simulations” help elucidate what would have been the necessary biophysical requirements for the root to maintain its functional, graded morphogen distribution by exploiting some alternative gradient-forming mechanisms to the one experimentally found. We have shown that in all cases, a trade-off occurs between space- and time-scales. In the evolutionary context, such theoretical explorations allow us to draw a better understanding on what sort of constraints and fitness landscapes plants have been facing, while becoming multi-cellular and organising into relatively large, and extremely plastic developing organisms. By explicitly having asked here, “why not” going for other mechanisms, we have highlighted “why” the plant has ‘chosen’ an apparently alternative approach (i.e. the reflux-loop) for solving its positional information problem – or, said differently, why a fast diffusive molecule and the reflux-loop are so fundamental for having evolved plant-like development.

## Endnotes

^a^Note that while in animal development cell fate determination (i.e. what a cell will usually develop into) is more strictly set up during embryogenesis, the plasticity presented by plants reveals a much less restricted cell fate in which regulated steps in the cell differentiation (i.e. the trajectory from progenitor to progeny) are more important.

^b^When besides transport also decay is taken into account, with a decay rate given by *d* (i.e. when Eq. 8a is extended to $\frac{dC_{n}}{\mathrm{dt}}=(p+q)C_{n-1}+qC_{n+1}-(p+2q)C_{n}-dC_{n}$, with the auxin level within the source cell 0 fixed at _*C*0_), the ratio between consecutive cells becomes $R_{n}=\frac{C_{n}}{C_{n-1}}=\frac{p+q}{q+d\left(1+\overset{L}{\underset{i=n+1}{\sum }}\pi_{j=n+1}^{i}R_{j}\right)}$, where *L* is the last cell in the cell file. Given that the decay *d* will typically be small compared to transport rates, also when decay is taken into account the ‘heaping up’ at the ‘dead end’ still presents the ratio $R_{n}\approx \frac{p+q}{q}$, but only as long as *L*−*n*is not too large, i.e. only sufficiently close to the ‘dead end’. The main difference to the pattern without decay is that far from the dead end (close to the source) the pattern becomes influx-driven, due to the fact that most of the breakdown takes place within the heaping-up region. Mathematically, when the cell file is sufficiently long, for *n*≪*L* the ratio can be approximated as $R_{n}\approx \frac{p+d+2q-\sqrt{{\left(p+d\right)}^{2}+4\mathrm{dq}}}{2q}$. When *q*≪*p*, this can be further simplified to $R_{n}\approx \frac{p}{p+d}$. The transition between the two regimes is very steep and takes place just within just a few cells (a property which does not depend on the specific parameter choices). In short, when not only transport, but also decay is taken into account, two distinct regions within the cell file will arise: an influx-driven part, presenting a very shallow exponential decrease (with $\lambda \approx 1/\log\left(\left(p+d\right)/p\right)$, followed by a heaping-up-driven part, presenting a very steep exponential increase (with $\lambda \approx 1/\log\left(\left(p+q\right)/q\right)$.

## Competing interest

The authors declare that they have no competing interests.

## Author’s contributions

VAG and AFMM conceived and designed the models and wrote the paper. VAG, BS, PH, AFMM discussed and interpreted the results and commented on the manuscript. All authors read and approved the final manuscript.
